# Bristol girls dance project (BGDP): protocol for a cluster randomised controlled trial of an after-school dance programme to increase physical activity among 11–12 year old girls

**DOI:** 10.1186/1471-2458-13-1003

**Published:** 2013-10-24

**Authors:** Russell Jago, Mark J Edwards, Simon J Sebire, Ashley R Cooper, Jane E Powell, Emma L Bird, Joanne Simon, Peter S Blair

**Affiliations:** 1 Centre for Exercise, Nutrition and Health Sciences, School for Policy Studies, University of Bristol, Bristol, UK; 2 Health and Social Sciences, University of the West of England, Bristol, UK; 3 School of Social and Community Medicine, University of Bristol, Bristol, UK

**Keywords:** Adolescent, Physical activity, Dance, Intervention, After-school

## Abstract

**Background:**

Many children do not meet current UK physical activity (PA) guidelines. Girls are less active than boys throughout childhood, and the age-related decline in PA, particularly from early adolescence, is steeper for girls than for boys. Dance is the favourite form of PA among UK secondary school aged girls. Delivering dance sessions after school could make a significant contribution to girls’ PA. Therefore, after-school dance sessions may be an appropriate and cost-effective activity through which adolescent girls’ PA levels can be increased.

**Design:**

Two-arm cluster randomised control trial and economic evaluation conducted in 18 secondary schools across the greater Bristol area. All Year 7 girls in participating schools will receive a 'taster’ dance session and subsequently be invited to participate in the project. There is space for up to 33 girls to participate in each school. Schools will be randomly assigned in equal numbers to intervention or control arms after baseline data has been collected. The nine intervention schools will receive a 20 week after-school dance-based intervention, consisting of 40 × 75 minute sessions, delivered by external dance instructors. Control schools will not receive the dance intervention. All measures will be assessed at baseline (time 0), at the end of the intervention period (time 1) and six months after the intervention has ended (time 2). Our primary interest is to determine the effectiveness and cost-effectiveness of the intervention to affect the objectively-assessed (accelerometer) mean weekday minutes of moderate-to-vigorous PA (MVPA) accumulated by Year 7 girls one year after the baseline measurement (time 2).

**Discussion:**

This paper describes the protocol for the Bristol Girls Dance Project cluster randomized controlled trial and economic evaluation, which is attempting to increase MVPA among Year 7 girls in UK secondary schools.

**Trial registration:**

ISRCTN52882523.

## Background

Physical activity (PA) is associated with lower levels of a number of health-based risk factors including insulin, glucose, blood pressure, body mass and is also associated with improved emotional well-being and self-esteem among young people [1]. Despite the benefits of regular PA, many young people do not meet the current UK recommendation of an hour of PA on most days of the week [2]. PA levels decline during childhood, with the start of secondary school a key period of change [3]. Girls are less active than boys throughout childhood and the age-related decline in PA, particularly from early adolescence, is steeper for girls than boys [3].

Organised after-school PA programmes that focus on increasing PA opportunities for a wide group of adolescents could be an effective means of engaging inactive adolescents in PA [4]. A systematic review reported that there had been five evaluations of after-school PA interventions that had employed objective evaluation methods [5]. Of the five studies, three interventions reported positive effects on PA while a fourth pilot study reported a trend towards increased PA when compared to the control group. Four of the five interventions were well received by the children and their parents. With the exception of one study conducted in Spain [6], all studies were conducted in the USA. Thus, although many UK secondary schools offer organised after-school PA programmes, a rigorous and systematic evaluation of this type of intervention has not been conducted.

Interventions that have been based on psychological theory have been more successful than those that have not, and psychological theories can provide key advances for intervention design as they facilitate the identification of key mediators and mechanisms of behaviour change [7]. Self-determination theory (SDT) [8] may be particularly appropriate for understanding adolescents’ PA [9] because it focusses on understanding the quality of individuals’ motivation (e.g. how self-determined their reasons for PA are). SDT contends that finding ways in which adolescents can develop a sense of choice and ownership over their own PA (autonomy), feel competent engaging in PA (competence), and feel supported within a broader social context (relatedness) will foster more self-determined forms of motivation (e.g. participating for fun or personally valued benefits) which are in turn positively associated with PA [8,10]. SDT therefore suggests that PA interventions which target enjoyable and valued activities and foster perceptions of ownership, competence and belonging, are more likely to result in a sustained behaviour change.

Dance is the favourite form of PA among UK adolescent girls [11] and is a desirable activity in which they can engage [12]. Dance overcomes many barriers to adolescent girls’ participation in PA as it is usually group-based (less likely to lead to public display and offers social interaction), non-competitive, and usually takes place indoors (not affected by weather). Dance therefore provides an appropriate medium through which to increase girls’ PA and apply SDT as it is has the potential to be intrinsically motivating and build girls’ perceived autonomy, competence and relatedness. Many girls who would normally drop out of other forms of PA during secondary school will engage in dance when it is available [13].

The applicability of a UK dance-based intervention to increase PA has not been tested. Delivering dance sessions after school, and focussing on increasing girls’ desire to engage in dance and their ability to take part with or without adult instruction, could make a significant contribution to girls’ PA levels.

### Feasibility trial

The Bristol Girls Dance Project (BGDP) feasibility study [14] was a three-arm, parallel group, cluster randomized controlled pilot trial and economic evaluation, with schools as the unit of allocation. Seven secondary schools were recruited and all Year 7 girls who were physically able to participate in Physical Education (PE) classes were invited to participate. For practical reasons the sample was limited to 30 girls per school. Three intervention schools received two 90-minute after-school dance classes per week, for nine weeks. Following extensive formative work [12,14-16], the sessions were based on hip-hop and street dance genres. All participants were asked to wear an Actigraph accelerometer for seven days at baseline (Week 0), during the last two weeks of the intervention (Week 8 or 9) and 3 months after the intervention ended (Week 20). The feasibility trial demonstrated that it is possible to recruit Year 7 girls and record the cost of the programme. An embryonic resource-use checklist was developed for use in the main trial economic evaluation. We also showed that girls would attend the dance sessions and it was feasible to collect PA data from the girls at three time points. The feasibility work suggested that it would be possible to achieve a mean increase of 10 additional minutes of MVPA per weekday (i.e. 50 minutes per week) if the session intensity was increased and inactive creative time reduced.

Evidence of cost-effectiveness is important for knowing where to invest scarce resources and commission programmes to maximise health outcomes in the population [17,18]. However, gathering the evidence is a challenge [19] where behaviour change is associated with health outcomes that have wider cultural and environmental determinants [20,21]. The feasibility trial demonstrated that it was possible to cost the dance programme, but the cost-effectiveness was not ascertained.

### Aims of the current study

The current study builds on the feasibility trial by examining the effect of a dance intervention on the MVPA levels of Year 7 girls. The specific research aims of the BGDP trial are as follows:

#### ***Primary aim***

1. Determine the effectiveness of the BGDP intervention to improve the objectively-assessed (accelerometer) mean weekday minutes of MVPA accumulated by Year 7 girls one year after the baseline measurement (T2 = time 0 + 52 weeks).

#### ***Secondary aims***

2. Determine the effectiveness of the BGDP intervention to improve the following secondary outcomes among Year 7 girls at T2:

a) Mean weekend day minutes of MVPA;

a) Mean weekday accelerometer counts per minute (providing an objective measure of the volume of overall PA in which participants engage);

a) Mean weekend day accelerometer counts per minute;

a) The proportion of girls meeting the recommendation of 60 minutes of MVPA per day;

a) Mean accelerometer-derived minutes of weekday sedentary time;

a) Mean EQ-5D-Y scores (EuroQol 5D Youth survey - a standardised instrument for measuring health outcomes);

a) Programme costs (school level).

3. Determine the effectiveness of the BGDP intervention during the intervention period (weeks 19–20 of the intervention – first follow-up) on all primary and secondary outcome variables.

4. Determine the extent to which any effects on primary and secondary outcomes are mediated by autonomous and controlled motivation towards PA and perceptions of autonomy, competence and relatedness in PA [8].

5. Determine the cost-effectiveness/utility of the intervention from a public sector perspective over the time frame of the study.

## Design

BGDP is a two-armed cluster randomised control trial in 18 secondary schools. The trial includes process, economic, quantitative and qualitative evaluations. The 18 schools will be recruited from state secondary schools (excluding Special Educational Needs, dance academies and privately/independently funded schools) operating within three Local Authorities: Bristol City Council, North Somerset Council, and Bath and North East Somerset Council. We aim to recruit up to 33 Year 7 girls from each school, with a minimum of 25 participants in each (450–594 participants overall). Schools must have at least 30 Year 7 girls, and be able and willing to allocate space for two after-school sessions per week for 20 weeks.

All schools fulfilling the inclusion criteria will be invited to participate and the first 18 schools that agree to participate will be enrolled. Additional schools will be placed in a reserve pool. If fewer than 25 girls are recruited in a given school, we will recruit a different school. Nine schools will be randomly assigned to the intervention arm and nine to the control arm.

### Participant recruitment

Following school recruitment, a participant recruitment campaign will be initiated in all 18 schools. A taster session will be provided for all Year 7 girls who are able to engage in PE classes. The sessions will be delivered by an external dance instructor. At the end of the taster session students will be told about the study (including details of the randomisation and data collection commitments). All girls will be provided with information packs for themselves and their parents, and will be asked to return informed consent forms. If more than 33 consent forms are returned in each school, 33 girls will be randomly selected to participate using a computer-generated algorithm. If a girl drops out of the study prior to baseline data collection she will be replaced by the first randomly chosen reserve, with this process repeated as necessary. No replacements will be made after baseline collection. All participants will receive a £10 gift voucher on completion (return of accelerometer) of each of the data collection phases (£30 in total). The study has been granted ethical approval from the funder, sponsor and the School for Policy Studies ethics committee at the University of Bristol. Written informed parental consent will be obtained for all participants.

### Sample size

Sample size calculations were performed to detect a mean difference of ten minutes of weekday MVPA between the intervention and control groups. The un-inflated sample size required for analysis to detect a difference of 10 minutes/day MVPA - assuming a standard deviation of 18 minutes [14] with 90% power and 5% two-sided alpha is 68 per arm. We estimated the 95% Confidence Interval (CI) for the school-associated Intra-class Correlation (ICC) in the pilot study to be < 0.001 to 0.087. If we assume that 20% of participants will not provide primary outcome data, the mean cluster size for analysis will be 24, resulting in a design effect of 3.0 using the upper 95% confidence limit for ICC. Thus, we will recruit a total of 18 schools and at least 450 girls.

### Randomisation

Randomisation will occur at the school level after baseline data has been collected. Balance between trial arms will be achieved with respect to Local Authority membership, mean minutes of participant MVPA at baseline, school size, and deprivation. Deprivation will be assessed as the percentage of pupils in the school eligible for the Department of Education’s Pupil Premium (additional funding given to schools to support disadvantaged pupils and bridge the attainment gap between them and their peers).

### Intervention description

Schools randomised to the intervention arm will receive a 20-week dance intervention, consisting of 2 × 75 minute after-school sessions per week (40 sessions in total), running between January and June 2014. Dance sessions will be led by an external dance instructor who will deliver a standardised programme which was developed in the feasibility trial. Instructors will attend a training programme before the intervention begins and a 'booster session’ after the first term of delivery. The dance programme focuses on building participants’ perceived autonomy to be active and perceived dance competence in a social, autonomy-supportive environment. The programme provides exposure to a wide range of dance styles. Participants in intervention schools will each be given a 'dance diary’ which they will be encouraged to complete between sessions. The diaries will help children to reflect on their learning and encourage them to set their own goals.

### Control school provision

Schools in the control arm will not receive the dance intervention and will continue with their normal schedule. Control schools will receive a £500 donation to the general school fund once all data has been collected from participants.

### Measures

Data will be collected from all participants (intervention and control) at three time-points.

1. Time 0 (T0) (baseline), September-November 2013.

2. Time 1 (T1) (baseline +19-20 weeks), June 2014.

3. Time 2 (T2) (baseline + 52 weeks), September-November 2014.

The following measures will be measured at each time point: 1) accelerometer-assessed PA; 2) self-completed psychosocial questionnaire containing variables that we hypothesise to function as mediators (including self-esteem measures); 3) self-completion of the EQ-5D-Y health questionnaire, and; 4) height and weight. Details of each measurement are outlined below. In addition at T0, for descriptive purposes, all parents will be asked to report their home postcode; which will be used to estimate the index of multiple deprivation for the primary residence. Girls will also be asked to self-report their level of dance experience using categories of 'none’, 'some’ or 'a lot’.

### Accelerometer assessed PA

Participants will wear an Actigraph GT3X+ accelerometer for seven days. Periods of ≥60 minutes of zero values will be defined as accelerometer’non-wear’ time and discarded. Participants will be included in the analysis if they provide ≥3 days (weekday or weekend) of data with at least 500 minutes of data between 06:00 and 23:00.

Mean minutes of daily MVPA will be established using the threshold developed by Evenson et al. [22], which has been shown to be an accurate threshold for this age group [23]. The following accelerometer variables will be derived:

#### ***Primary outcome***

1. Mean MVPA on weekdays a year after baseline measurement (T2).

#### ***Secondary outcomes***

2. Mean weekend day minutes of MVPA (T1 and T2);

3. Mean weekday minutes of MVPA (T1 and T2);

4. Mean weekday accelerometer counts per minute (providing an indication of the volume of activity in which the girls engage) (T1 and T2);

5. Mean weekend day accelerometer counts per minute (T1 and T2);

6. Proportion of girls meeting the recommendation of 60 minutes of MVPA per day (T1 and T2);

7. Mean minutes of sedentary time per weekday (≤100 counts per minute) (T1 and T2).

### Psychosocial questionnaire

All participants will be asked to complete a 66 item questionnaire at each time point. The questionnaire, which will be programmed onto a tablet computer, will assess psychosocial variables that could be influenced by the intervention and/or mediate the effect of the intervention on MVPA. Aligned with SDT, autonomous (8 items) and controlled motivation (7 items) [24] and perceptions of autonomy (6 items), competence (6 items) and relatedness (5 items) [25,26] within PA will be measured. Self-esteem (9 items) [27] will also be measured. All measures were piloted in the feasibility trial [14] and displayed evidence of internal consistency among Year 7 girls. Following reverse scoring of negatively worded items, subscale mean scores will be calculated.

**Figure 1:**
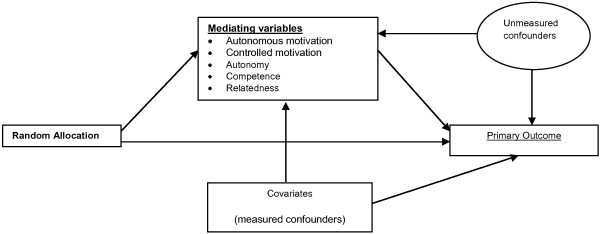
Hypothesised mediation model.

As shown in Figure 1, we hypothesise that autonomous and controlled motivation for PA, and perceptions of autonomy, competence and relatedness in PA will mediate the effect of the intervention on weekday MVPA.

### Costs/economic outcome data

The EuroQol 5D Youth survey (EQ-5D-Y), validated for use in children and adolescents [28], will be applied as a secondary outcome measure of health related quality of life in the trial. The EQ-5D-Y questionnaire is a standardised instrument for measuring health outcomes in youth. Public sector resources used at each stage of programme delivery will be recorded retrospectively using the resource use checklist tool developed during the feasibility study. Time sheet, expenses and travel data records will all be utilised. Costs will be estimated using the checklist tool developed for this purpose and prices from published or established sources. Costs will be uprated in line with inflation to 2014–15 prices.

### Process evaluation

A process evaluation will be conducted in the nine intervention schools. The process evaluation will report on consent, recruitment, attendance and adherence rates. The dose of the intervention (i.e. number of planned sessions delivered) will be recorded for each school. Intervention fidelity will be assessed by: a) dance instructors’ completion of a log-book indicating whether they delivered planned core components of each session (*fully*, *partially* or *not at all*); and b) observation/audio recording of four random sessions delivered by each dance instructor. Observers will rate the degree to which the instructors delivered the core components of the session. Audio recordings will be rated using a validated tool [29], to measure the extent to which dance teachers’ teaching style was autonomy-supportive. At the end of the four observed sessions in each school, participants will be asked to complete a perceived exertion [30] and enjoyment [31] questionnaire.

To assess any contamination of the control group from dance classes locally, we will collect data on extra-curricular provision (including dance) in all 18 schools. School-level data will be collected from school staff at each measurement point. Additionally, girls will be asked if they attend dance classes outside of school at each measurement point. Any girls who withdraw from the intervention will be asked to complete an exit questionnaire to explore their reasons for withdrawal.

### Statistical analysis

The analysis and presentation of the trial will be in accordance with CONSORT guidelines, with the primary comparative analyses being conducted on an intention-to-treat (ITT) basis and due emphasis placed on confidence intervals for the between-arm comparisons. To take appropriate account of the hierarchical nature of the data, we will use multivariable mixed effects linear regression to estimate difference in the primary outcome for intervention group versus control, adjusting for baseline MVPA and randomisation variables. In a secondary analysis, we will further adjust for variables that were imbalanced between the trial arms at baseline. To assess the potential effect of missing data on the outcomes, in sensitivity analyses we will impute data using standard multiple imputation approaches.

We will investigate the effect of adherence to the intervention using instrumental variable regression, with adherence defined as girls attending 25 of the 40 sessions. Appropriate interaction terms will be entered into the primary regression analyses for mean weekday MVPA in order to conduct pre-specified subgroup analyses that will include baseline level of dance experience ('none’, 'some’ or 'a lot’), socioeconomic position (based on the index of multiple deprivation for the home postcode – continuous variable), and baseline weekday minutes of MVPA (continuous variable). Since the trial is powered to detect overall differences between the groups rather than interactions of this kind, these analyses are considered exploratory and results will be presented using confidence intervals and interpreted with due caution.

We will explore whether the effect of the intervention on the primary and secondary outcomes is mediated by autonomous and controlled motivation for PA and/or perceptions of autonomy, competence and relatedness need satisfaction in PA. This will be achieved by methods based on the processes of Emsley and colleagues [32].

Quantitative process evaluation data (e.g. attendance rates) will be analysed using appropriate descriptive statistics for normally distributed variables (using the mean and standard deviation) and variables without such a distribution (using the median and inter-quartile ranges). Ratings of instructor's teaching styles will be made from audio recordings combined with real time observation notes. Each item will be rated in every 5 minute lesson period, these values will be summed and divided by the number of five-minute intervals in the lesson. Item scores will be summed to provide quantitative scores for five teaching elements; Relatedness support, Structure before the activity, Structure during the activity, autonomy support and controlling teaching behaviour.

### Economic analyses

Economic analysis will be set within a cost 'effectiveness’ framework. The mean incremental costs associated with intervention delivery at the school level will be estimated from a public sector perspective [14]. Public sector costs will be related to incremental change in accelerometer-derived MVPA minutes per weekday and incremental change in EQ-5D-Y scores to estimate cost per minute of MVPA, cost per Quality-adjusted Life Year (QALY), average cost per school and average cost per user, with the associated CIs reported. EQ-5D-Y will be administered at T0, T1 and T2. We will also extend the balance sheet framework to include the proportion of participants meeting the 60-minute per day MVPA recommendation. Programme costs will also be determined at the school level.

Confidence intervals for the *incremental cost-effectiveness ratio* (ICER) will be calculated using bootstrapping at the school level. If appropriate we will produce cost-effectiveness acceptability curves for a range of thresholds and conduct threshold analysis to compare our cost per QALY estimates with National Institute for Health and Care Excellence (NICE) benchmark values. The time frame for the health economic evaluation is the length of the study, as the focus is to consolidate learning from our feasibility study in a full trial, and not to attempt to make long term predictions of cost-effectiveness at this stage. Uncertainty will be explored using sensitivity analyses and findings will be presented for a range of decision makers at the societal and funder levels, including Local Authorities and the National Health Service (NHS), from a public sector perspective.

### End of study qualitative assessment

Qualitative methods will be used at the end of the intervention to ascertain elements of the intervention that worked well, potential improvements, and factors that might affect future dissemination/roll-out.

A focus group will be conducted with a selection of participants at all nine intervention schools. Participants will be purposively sampled to represent a range of attendance levels, with approximately 6–8 participants per group. The focus groups will address facilitators, barriers to participants’ engagement, perceived impact and their views on promoting the dance project for a larger roll-out.

Semi-structured telephone interviews will be conducted with all dance instructors, addressing their experiences of delivering the intervention, barriers and facilitators, and factors central to supporting their continued involvement if the programme was implemented more widely. Semi-structured interviews will also be conducted with the primary contact at each intervention school, focusing on the logistical and organisational factors affecting delivery within school and how best to market the project for wider implementation. Interviews and focus group recordings will be transcribed verbatim and analysed via NVivo software, using thematic analysis [33].

### Current status of trial (23/09/2013)

18 schools have been recruited, with four reserves. Dance instructors have been recruited to deliver the taster and after-school dance sessions. Baseline data is currently being collected. Randomisation of schools to control and intervention arms will be conducted in October 2013, and schools will be informed of their arm allocation soon after. The after-school dance sessions will be delivered between January and June/July 2014 in the nine intervention schools.

## Discussion

This paper describes the rationale and methods that will be used for the BGDP cluster randomised controlled trial. The trial is attempting to increase levels of PA amongst Year 7 girls in the greater Bristol area, UK.

## Abbreviations

BMI: Body mass index; CI: Confidence interval; CONSORT: Consolidated standards of reporting trials; EQ-5D-Y: EuroQol 5D youth survey; ICC: Intra-class correlation; ICER: Incremental cost-effectiveness ratio; ITT: Intention-to-treat; MVPA: Moderate to vigorous physical activity; NHS: National Health Service; NICE: National Institute for Health and Care Excellence; PA: Physical activity; QALY: Quality adjusted life year; SDT: Self-determination theory.

## Competing interests

The authors declare that they have no competing interests.

## Authors’ contributions

RJ, SS, AC, and JP conceived the pilot study and conducted the formative work and its analysis. RJ is the Principal Investigator and grant holder. ME is the Trial Manager. SS developed the process evaluation plan and contributed towards the qualitative and quantitative analyses. AC contributed to the design of the study and the writing of the manuscript. JP contributed to the design of the study and, along with EB, developed the economic evaluation plan. PB is the trial statistician and contributed to the statistical analyses plan. JS is the Senior Research Manager and has contributed to the study design. The first draft of this manuscript was produced by RJ, ME, SS and JP. All authors have critically reviewed the paper and approved its submission.

## Pre-publication history

The pre-publication history for this paper can be accessed here:

http://www.biomedcentral.com/1471-2458/13/1003/prepub

## References

[B1] Parfitt G Eston RG The relationship between children’s habitual activity level and psychological well-being Acta Paediatr 2005 13 12 1791 1797 10.1080/08035250500268266 16421041

[B2] Department of Health, Health Improvement and Protection Start active, stay active: a report on physical activity from the four home countries’ chief medical officers 2011 London: Department of Health

[B3] Nader PR Bradley RH Houts RM McRitchie SL O’Brien M Moderate-to-vigorous physical activity from ages 9 to 15 years JAMA 2008 13 3 295 305 10.1001/jama.300.3.295 18632544

[B4] Jago R Baranowski T Non-curricular approaches for increasing physical activity in youth: a review Prev Med 2004 13 1 157 163 10.1016/j.ypmed.2004.01.014 15207997

[B5] Pate RR O’Neill JR After-school interventions to increase physical activity among youth Br J Sports Med 2009 13 1 14 18 19019903 10.1136/bjsm.2008.055517

[B6] Vizcaino VM Aguilar FS Gutierrez RF Martinez MS Lopez MS Martinez SS Garcia EL Artalejo FR Assessment of an after-school physical activity program to prevent obesity among 9- to 10-year-old children: a cluster randomized trial Int J Obesity 2008 13 12 22 10.1038/sj.ijo.0803738 17895883

[B7] Baranowski T Jago R Understanding mechanisms of change in children’s physical activity programs Exerc Sport Sci Rev 2005 13 4 163 168 10.1097/00003677-200510000-00003 16239832

[B8] Ryan RM Deci EL Self-determination theory and the facilitation of intrinsic motivation, social development, and well-being Am Psychol 2000 13 1 68 78 11392867 10.1037//0003-066x.55.1.68

[B9] Rosenkranz RR Lubans DR Peralta LR Bennie A Sanders T Lonsdale C A cluster-randomized controlled trial of strategies to increase adolescents’ physical activity and motivation during physical education lessons: the motivating active learning in physical education (MALP) trial BMC Public Health 2012 13 834 10.1186/1471-2458-12-834 23025261 PMC3524026

[B10] Ntoumanis N A self-determination approach to the understanding of motivation in physical education Br J Educ Psychol 2001 13 225 242 10.1348/000709901158497 11449934

[B11] O’Donovan TM Kay TA Focus on girls in sport Br J Teach Phys Educ 2005 13 1 29 31

[B12] Jago R Davis L McNeill J Sebire SJ Haase A Powell J Cooper AR Adolescent girls' and parents’ views on recruiting and retaining girls into an after-school dance intervention: Implications for extra-curricular physical activity provision Int J Behav Nutr Phys Act 2011 13 1 91 10.1186/1479-5868-8-91 21861892 PMC3177760

[B13] Quin E Redding E Frazer L Dance science report: the effects of an eight week creative dance programme on the physiological and psychological status of 11–14 year old adolescents 2007 Hampshire: Hampshire Dance and LABAN 1 3

[B14] Jago R Sebire SJ Cooper AR Haase AM Powell J Davis L McNeill J Montgomery AA Bristol girls dance project feasibility trial: outcome and process evaluation results Int J Behav Nutr Phys Act 2012 13 83 10.1186/1479-5868-9-83 PMC3411449 22747608

[B15] Sebire SJ McNeill J Davis L Haase AM Powell J Cooper AR Powell RJ Designing extra-curricular dance programmes: UK physical education and dance teachers’ perspectives Open J Prev Med 2013 13 1 111 117 10.4236/ojpm.2013.31014

[B16] Jago R Jonker ML Missaghian M Baranowski T Effect of 4 weeks of Pilates on the body composition of young girls Prev Med 2006 13 3 177 180 10.1016/j.ypmed.2005.11.010 16376979

[B17] Buchanan J Wolstenholme J Foster C A rapid review of economic literature related to the promotion of physical activity, play and sport for pre-school and school-age children in family, pre-school, school and community settings 2008 London: NICE

[B18] Kelly MP McDaid D Ludbrook A Powell JE Economic appraisal of public health interventions 2005 London: Health Development Agency

[B19] Richardson AK Investing in public health: barriers and possible solutions J Public Health (Oxf) 2012 13 3 322 327 10.1093/pubmed/fds039 22696599

[B20] Ogilvie D Bull F Powell J Cooper AR Brand C Mutrie N Preston J Rutter H iConnect C: an applied ecological framework for evaluating infrastructure to promote walking and cycling: the iConnect study Am J public health 2011 13 3 473 481 10.2105/AJPH.2010.198002 21233429 PMC3036680

[B21] Powell J Compression of morbidity outcomes key to investment in public health J Public Health (Oxf) 2012 13 3 329 10.1093/pubmed/fds043 22615418

[B22] Evenson KR Catellier DJ Gill K Ondrak KS McMurray RG Calibration of two objective measures of physical activity for children J Sports Sci 2008 13 14 1557 1565 10.1080/02640410802334196 18949660

[B23] Trost SG Loprinzi PD Moore R Pfeiffer KA Comparison of accelerometer cut-points for predicting activity intensity in youth Med Sci Sports Exerc 2011 13 7 1360 1368 10.1249/MSS.0b013e318206476e 21131873

[B24] Markland D Tobin V A modification of the behavioral regulation in exercise questionnaire to include an assessment of amotivation J Sport Exer Psychol 2004 13 191 196

[B25] McAuley E Duncan T Tammen VV Psychometric properties of the Intrinsic Motivation Inventory in a competitive sport setting: a confirmatory factor analysis Res Q Exerc Sport 1989 13 1 48 58 10.1080/02701367.1989.10607413 2489825

[B26] Standage M Duda JL Ntoumanis N A test of self-determination theory in school physical education Br J Educ Psychol 2005 13 Pt 3 411 433 16238874 10.1348/000709904X22359

[B27] Marsh HW Sydney UoW Self description questionnaire (SDQ) II: a theoretical and empirical basis for the measurement of multiple dimensions of adolescent self-concept. A test manual and research monograph University of Western Sydney 1992 New South Wales: Faculty of Education

[B28] Wille N Badia X Bonsel G Burstrom K Cavrini G Devlin N Egmar AC Greiner W Gusi N Herdman M Development of the EQ-5D-Y: a child-friendly version of the EQ-5D Qual life res Int J Qual Life Aspects Treat Care Rehab 2010 13 6 875 886 10.1007/s11136-010-9648-y PMC2892611 20405245

[B29] Haerens L Aelterman N Van den Berghe L De Meyer J Soenens B Vansteenkiste M Observing physical education teachers’ need-supportive interactions in classroom settings J Sport Exerc Psychol 2013 13 1 3 17 23404876 10.1123/jsep.35.1.3

[B30] Robertson RJ Goss FL Boer NF Peoples JA Foreman AJ Dabayebeh IM Millich NB Balasekaran G Riechman SE Gallagher JD Children’s OMNI scale of perceived exertion: mixed gender and race validation Med Sci Sports Exerc 2000 13 3 452 458 10694131 10.1097/00005768-200002000-00029

[B31] Macfarlane D Kwong WT Children’s heart rates and enjoyment levels during PE classes in Hong Kong primary schools Ped Exerc Sci 2003 13 179 190

[B32] Emsley R Dunn G White IR Mediation and moderation of treatment effects in randomised controlled trials of complex interventions Stat methods Med Res 2010 13 3 237 270 10.1177/0962280209105014 19608601

[B33] Braun V Clarke V Using thematic analysis in psychology Qual Res Psychol 2006 13 77 101 10.1191/1478088706qp063oa

